# Computed tomography perfusion in predicting radiation therapy response in dogs and a cat with head and neck tumors

**DOI:** 10.1093/jvimsj/aalaf025

**Published:** 2026-01-21

**Authors:** Soyeon Kim, Eunjee Kim, Min-Ok Ryu, Kyoungwon Seo, Junghee Yoon, Jihye Choi

**Affiliations:** Department of Veterinary Medical Imaging, College of Veterinary Medicine, Seoul National University, Seoul 08826, South Korea; Department of Veterinary Medical Imaging, College of Veterinary Medicine, Seoul National University, Seoul 08826, South Korea; Laboratory of Veterinary Internal Medicine, Department of Veterinary Clinical Science, College of Veterinary Medicine, Seoul National University, Seoul 08826, South Korea; Laboratory of Veterinary Internal Medicine, Department of Veterinary Clinical Science, College of Veterinary Medicine, Seoul National University, Seoul 08826, South Korea; Department of Veterinary Medical Imaging, College of Veterinary Medicine, Seoul National University, Seoul 08826, South Korea; Department of Veterinary Medical Imaging, College of Veterinary Medicine, Seoul National University, Seoul 08826, South Korea

**Keywords:** cats, computed tomography, dogs, radiotherapy, tumor perfusion

## Abstract

**Background:**

Predicting tumor response to radiation therapy (RT) is challenging, as conventional assessment primarily considers morphology. Computed tomography (CT) perfusion might provide early prediction by evaluating tumor oxygenation and vascularity.

**Hypothesis/Objectives:**

To evaluate the association between CT perfusion variables and tumor size reduction, and their potential to predict outcomes after RT.

**Animals:**

Eleven dogs and 1 cat with malignant head and neck tumors, including squamous cell carcinoma, melanoma, and adenocarcinoma.

**Methods:**

This retrospective study evaluated CT perfusion and tumor size before RT, and at 0, 30, and 90 days after RT. Perfusion variables, including blood flow (BF), blood volume (BV), and flow extraction (FE), were measured as absolute values and normalized to contralateral tissue (rc) and muscle (rm). Changes were compared with the previous (pΔ) and following (fΔ) examinations.

**Results:**

Median values before RT were BV 29.7 mL/100 mL, BF 229.9 mL/min/100 mL, and FE 39.5 mL/min/100 mL. Tumor volumes were generally smaller after RT. The perfusion variables increased at day 30, and subsequently declined by day 90. The rcBV and early increases in BF (pΔBF) were associated with change in tumor size. Higher BF or BV before RT correlated with greater tumor reduction. The change in rmBV relative to the previous scan (pΔrmBV) also correlated with sequent tumor size changes.

**Conclusions and clinical importance:**

Computed tomography perfusion could offer functional biomarkers for early RT response assessment. Higher BF and BV before RT were associated with better outcomes, and normalized variables, particularly rcBV and pΔBF more reliably reflected tumor size changes than absolute values.

## Introduction

Radiation therapy (RT) is widely used for treatment of head and neck tumors.^1–4^ Tumor response to RT varies widely depending on factors such as tumor type, location, and vascular characteristics.^5^^,^^6^ Traditional tumor response evaluation methods, such as the Response Evaluation Criteria in Solid Tumors (RECIST) primarily rely on reductions in tumor size.^7^ Since RECIST does not account for tumor biology, its effectiveness in predicting treatment response is limited.^8^^,^^9^ Therefore, one of the major challenges in veterinary radiation oncology is early prediction of treatment response to guide clinical decision.

One of the key determinants of RT efficacy is tumor oxygenation, as well-oxygenated tumors are more radiosensitive, while hypoxic tumors have greater radioresistance due to reduced formation of DNA-damaging free radicals, leading to treatment failure and recurrence.^10^^,^^11^ Since direct measurement of tumor oxygenation is challenging in veterinary medicine, perfusion imaging provides a practical, indirect assessment. As perfusion reflects blood flow (BF) supplying oxygen and nutrients to the tumor microenvironment, assessing tumor perfusion before and during RT can help predict treatment response and optimize therapeutic strategies.^12^

Computed tomography (CT) perfusion is a valuable non-invasive imaging tool for evaluating tumor vascular physiology and hemodynamics.^8^^,^^13–17^ Computed tomography perfusion allows for quantifying tumor perfusion variables, including BF, blood volume (BV), and permeability, which reflect tumor microcirculation and oxygenation.^13^^,^^14^^,^^17^^,^^18^ Computed tomography perfusion has been investigated as a potential imaging biomarker for early prediction of RT response in human medicine.^15^^,^^19–24^ Compared with RECIST, CT perfusion enables earlier identification of poor responders before significant morphological changes occur and might guide alternative therapeutic strategies.^7^^,^^9^^,^^22^^,^^25^^,^^26^ High BF, BV, and flow extraction (FE) correlate with better tumor response, likely due to enhanced tumor oxygenation although there are controversies.^21^^,^^22^^,^^26^^,^^27^

Despite its clinical utility in human oncology, few studies have evaluated its role in assessing tumor perfusion and predicting treatment response in dogs and cats and standardized imaging protocols and reference values are still limited.^1–3^^,^^28^^,^^29^ Given the increasing use of RT in veterinary medicine, a better understanding of clinical role of CT perfusion is essential for optimizing treatment strategies.

This study evaluated the CT perfusion features of malignant head and neck tumors and examined changes in perfusion variables after RT. In addition, the relationship between CT perfusion and tumor volume reduction was assessed. This study was hypothesized that tumors with higher pre-treatment BF and BV would show greater tumor size change after RT, and that relative perfusion variables, normalized to contralateral normal tissue, could provide more consistent results by reduced individual variability. The purpose of this study was to evaluate the potential role of CT perfusion as a functional imaging biomarker in veterinary oncology, with identifying early patterns of tumor response to RT and clinical outcomes in dogs and cats.

## Materials and methods

This study was approved by the Institutional Animal Care and Use Committee at Seoul National University, and animals were managed in accordance with the Guidelines for Animal Experiments of Seoul National University (SNU IACUC-SNU-230501-1-1).

### Case selection

This retrospective, preliminary study included client-owned dogs and cats with head and neck tumors that underwent CT perfusion imaging before and after RT at the Seoul National University Veterinary Medical Teaching Hospital between August 2023 and December 2024. Animals were included in the study if they met the following criteria: (1) a strongly suspected diagnosis based on cytology, histology, or characteristic CT and magnetic resonance imaging (MRI) findings as determined by a radiologist (J.H.C.) certified by the Korean College of Veterinary Medical Imaging, (2) the CT perfusion imaging performed during simulation CT for RT planning, and (3) at least one CT perfusion imaging before or after RT. Animals were excluded from the study if the animal previously received surgery, chemotherapy, or RT for the current tumor. Owner consent for diagnostic testing was obtained before inclusion.

### Clinical data

For all dogs and cats, data on treatment administered at each CT perfusion procedure, and systolic blood pressure and heart rate during CT perfusion were collected. Treatment information recorded included medications, such as non-steroidal anti-inflammatory drugs, anti-epileptic drugs, corticosteroids, and chemotherapy.

### Before and after RT timeline

Computed tomography perfusion imaging and tumor size evaluations using CT and MRI were performed before RT and at 0, 30, and 90 days after the completion of RT.

All procedures were performed under general anesthesia. After feed had been withheld for at least 8 h, a 22 or 24-gauge intravenous catheter was placed into the cephalic vein for anesthesia and contrast injection. The animals were premedicated with midazolam (0.2 mg/kg, Midazolam; Bukwang Pharmaceutical). Anesthesia was induced with alfaxalone (2 mg/kg, Alfaxan; Jurox) or with propofol (4 mg/kg; Daewon Pharm.) and maintained with isoflurane (2%-4%; Ifran, Hana Pharm) in 100% oxygen (1-2 L/min). During the anesthesia, the physiological variables were continuously monitored: blood pressure using an automatic non-invasive monitor (Vet25, SunTech Medical), body temperature with a manual thermometer (MT200, Microlife Corporation), oxygen saturation and heart rate with ECG tracing, and respiratory rate with a respiratory sensor.

Radiation therapy was administered using 6 MV photon beams delivered by a linear accelerator (Synergy, Elekta) equipped with a 160-micromultileaf beam collimator and an integrated cone-beam CT system for image-guided RT. All treatments were planned and delivered using volumetric modulated arc therapy techniques. The plans were generated with the treatment planning system (Monaco version 6.1.2.0, Elekta) utilizing a Monte Carlo algorithm to optimize dose distribution.

For target delineation, CT and MRI images were automatically fused in the Monaco treatment planning system using its integrated multimodal image registration tools. The gross tumor volume (GTV) was defined using a fusion of pre-contrast CT and post-contrast T1W MR images acquired after gadolinium injection. Margins for clinical target volume (CTV) was defined by adding microscopic disease extension regions to GTV. The planning target volume (PTV) was defined by expanding the CTV by 1-3 mm in all directions to account for potential microscopic tumor extension and setup uncertainties. Organs at risk (OAR), including the brain, spinal cord, cochlea, optic chiasm, optic nerves, eyes, lenses, lacrimal glands, salivary glands, and skin, were contoured on CT images, with additional structures delineated as necessary. The primary planning objective was to ensure coverage of at least 99% of the GTV and 95% of the PTV with the prescribed dose. The maximum dose outside the PTV was limited to 107% of the prescribed dose. Treatment plans utilized 1 or more continuously rotating coplanar arcs with beam collimation achieved using the micromultileaf beam collimator. Animals were immobilized in the same manner as during the simulation CT scans, and cone-beam CT imaging was performed before each treatment fraction to verify accurate positioning.

### Conventional CT and CT perfusion

Under general anesthesia, all cases were positioned in sternal recumbency using a modified, commercially available frameless stereotactic positioning device used for RT planning. The isocenter was set at the presumed center of the tumor. Computed tomography images were obtained with a 160-slice CT scanner (Aquilion Lightning 160 MODEL TSX-036A, Canon Medical System) using scanning variables as follows: tube voltage = 120 kVp, tube current = 200 mAs, rotation time = 1 s, pitch = 0.8, and slice thickness = 0.5 mm. A pre-contrast CT scan was performed from the nose to the cranial cervical vertebrae.

For CT perfusion, dynamic contrast-enhanced CT scan was conducted using the pre-contrast soft tissue reconstruction in a soft tissue window. A 4-cm field of view (FOV) (z-axis = 40 mm) was selected to include the center of the tumor as identified on the pre-contrast CT images. A 60-second continuous scan was performed with the initiation of the intravenous injection of iodinated contrast medium 300 mg I/mL iodine (Omnipaque, GE Healthcare) at a dose of 1 to 1.5 mL/kg body weight, administered using a power injector (Stellant, MEDRAD) at 2-3 mL/s. This was followed by a saline bolus flush of 1 mL/kg at the same injection rate. Images were obtained at 120 kV, 200 mA, 1 s rotation time, 0.5 mm scan slice thickness, 1.8 s time interval, and 0.5 mm reconstruction slice thickness.

Then, post-contrast CT images were obtained after additional 2 mL/kg of iodinated contrast medium, injected 2-3 mL/s. A post-contrast scan of the head was then conducted with the same scanning variables as the pre-contrast scan. Images were reconstructed with a slice thickness of 0.5 mm using both bone and soft tissue reconstruction algorithms.

### MRI scan

Magnetic resonance imaging scans were performed using a 1.5-T scanner (SIGNA Creator 1.5 T; GE Healthcare) with an 80-channel flex coil array. The dog was placed in sternal recumbency, and the imaging protocol included T2-weighted (T2W) and T1-weighted (T1W) sequences in dorsal, sagittal, and transverse planes; transverse plane of T2W-fluid-attenuated inversion recovery (FLAIR), and post-contrast T1W in dorsal, sagittal, and transverse planes. Post-contrast T1W was obtained after intravenous administration of a gadolinium-based contrast agent (0.01 mmol/kg, Dotarem; Guerbet). The scan FOV was selected for encompassing the whole tumor parenchyma and peri-lesional area in head and neck tumors.

### Tumor size assessments

Computed tomography images were reviewed using an image viewer (PACS, Infinitt Healthcare Co., Ltd.) by an observer (S.Y. K.), a PhD candidate in veterinary radiology. The images were evaluated using both a soft tissue window (window width: 200 HU; window level: 40 HU) and a bone window (window width: 4500 HU; window level: 450 HU).

Tumor size, including the length, width, height, and volume, was measured. The length (cranial-caudal axis), width (left–right axis), and height (ventral-dorsal axis) of the tumor were measured using multiplanar reconstructions, aligned with planes parallel and orthogonal to the head. Tumor volume was then calculated using the ellipsoid formula:


(1) \begin{align*}& \mathrm{Volume} = 4/3 \times \pi \times \mathrm{length} \times \mathrm{width} \times \mathrm{height}.\end{align*}

Tumor size measurements were performed on CT images acquired before RT and at follow-up time points on post RT days 0, 30, and 90. If the mass was not clearly defined on CT, measurements were conducted on post-contrast T1W MRI images using the same method described for CT. Tumor size change ratios were assessed relative to both previous and following examinations. Ratios compared to the previous examination were expressed as p**Δ***l*, p**Δ***w*, p**Δ***h*, and p**Δ***V* for length, width, height and volume, respectively. The ratios relative to the following examination were expressed as the following notations f**Δ***l*, f**Δ***w*, f**Δ***h*, and f**Δ***V*, representing the length, width, height, and volume, respectively. These variables were used to evaluate the temporal relationship between perfusion changes and tumor size variations during RT follow-up.

### CT perfusion analysis

**Figure 1:**
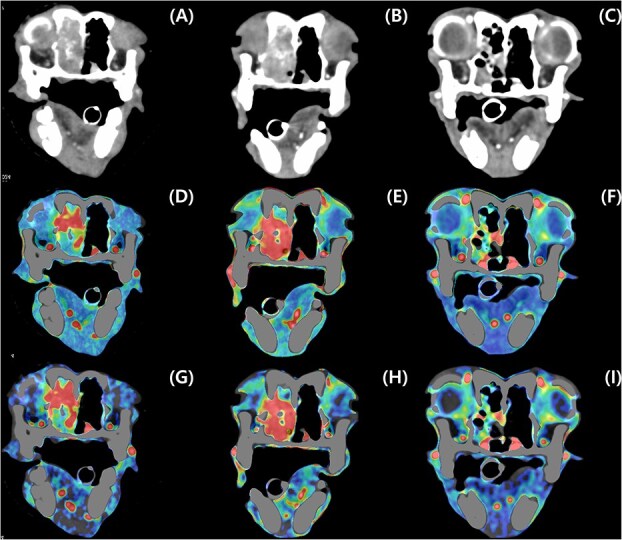
Contrast-enhanced transverse CT images of a dog with an adenocarcinoma treated with radiation therapy (RT). The tumor volume decreases from the images before RT (A, D, and G) through the day 0 (B, E, and H) and day 30 (C, F, and I) after RT. The tumor volume change (A-C) was compared with corresponding perfusion map of blood flow (mL/min/100 g) (D-F) and blood volume (mL/100 mL) (G-I).

Computed tomography perfusion was analyzed using a CT software based on a delay-insensitive deconvolution method (VITREA, Canon Medical Systems). In CT perfusion imaging, two regions of interest (ROIs) were placed on the same slice by a single author (S.Y.K.). One ROI for arterial inflow was positioned either on the deep lingual artery or on the carotid artery, depending on the primary tumor location. The other ROI was drawn circularly within the most enhancing region of the tumor parenchyma, ensuring maximal inclusion of representative tumor tissue. Then, the time-density curves derived from the ROIs generated perfusion maps (Figure 1) representing BV, BF, and FE. The BF and BV maps were made using the Maximum Slope algorithm, while the FE map was produced using the Patlak algorithm. Blood volume reflects the total amount of blood within the tissue volume and was expressed in mL/100 mL. BF, representing the rate of arterial BF into the tissue, was calculated based on the arterial input function and expressed in mL/min/100 mL. Flow extraction indicating vascular permeability and extraction efficiency, was quantified in mL/min/100 mL. Perfusion variable values were extracted from these color-coded maps based on the previously defined tumor ROIs. A detailed classification of perfusion variable measurements is provided in Table 1. Tumor perfusion variables were analyzed both as absolute values and as relative values. The relative values were obtained by normalizing the tumor variables to two reference regions: the corresponding contralateral normal parenchyma (rc) and normal muscle tissue (rm).

**Table 1:** Evaluation variables of head and neck tumors on CT perfusion study.

**Assessment**	**Category**	**Evaluation variables**
**Perfusion before RT**	Absolute values	Blood flow (BF), Equivalent blood volume (BV), and Flow extraction (FE)
	Relative to the contralateral parenchyma (rc)	rcBF, rcBV, and rcFE
	Relative to the contralateral muscle (rm)	rmBF, rmBV, and rmFE
**Change ratio compared to the previous examination after RT (pΔ)**	p**Δ** of absolute values	p**ΔBF,** p**ΔBV,** and p**ΔFE**
	p**Δ** of rc (p**Δrc)**	p**ΔrcBF,** p**ΔrcBV,** and p**ΔrcFE**
	p**Δ** of rm (p**Δrm)**	p**ΔrmBF,** p**ΔrmBV,** and p**ΔrmFE**
**Change ratio of following examination after RT (fΔ)**	f**Δ of** absolute values	f**ΔBF,** f**ΔBV,** and f**ΔFE**
	f**Δ of rc** (f**Δrc)**	f**ΔrcBF,** f**ΔrcBV,** and f**ΔrcFE**
	f**Δ of rm** (f**Δrm)**	f**ΔrmBF,** f**ΔrmBV,** and f**ΔrmFE**

Changes in CT perfusion and tumor size were assessed descriptively due to the small and heterogeneous sample population.

## Results

### Case demographics and clinical data

A total of 12 cases (11 dogs and 1 cat) met the inclusion criteria. They consisted of 8 neutered males and 4 neutered females. The breeds included 4 Maltese, 3 Poodles, 2 Schnauzers, and 1 each of following breeds: Dachshund, Cocker Spaniel, and Domestic Shorthair. The median age was 11.9 years (range, 7.2-16.9 years); and the median weight was 5.2 kg (range, 2.9-13 kg). Head and neck tumors included 6 squamous cell carcinomas (SCC), 3 melanomas, and 3 nasal adenocarcinomas. The primary tumor locations were as follows: nasal cavity (*n* = 3), mandible (*n* = 4), maxilla (*n* = 3), thyroid (*n* = 1), and tonsil (*n* = 1). Twelve cases received medications, including corticosteroids (*n* = 5), nonsteroidal anti-inflammatory drugs (*n* = 6), anti-epileptic drugs (*n* = 1), gabapentin (*n* = 3), antibiotics (*n* = 6), carboplatin as radiosensitizers (*n* = 4), gastrointestinal protectants (*n* = 4), analgesics (*n* = 3), and hepatoprotectants (*n* = 2).

### Tumor size before RT

There was no substantial difference of tumor volume among the tumor types. On images before RT, the median tumor width, height, and length were 2.7 cm (range, 0.9-7.6 cm), 2.3 cm (range, 0.8-5.1 cm), and 5.0 cm (range, 1.7-6.5 cm), respectively, with an overall median tumor volume was 12.7 cm^3^ (range, 0.6-65.5 cm^3^). Before RT, adenocarcinoma exhibited the largest median tumor volume, while melanoma had the smallest (Table 2).

**Table 2:** Tumor volume at before and after radiation therapy at different time points according to head and neck tumor types.

**Tumor types**	**Before-RT** **(*n* = 12)**	**After RT**		
		**Day 0** **(*n* = 7)**	**Day 30** **(*n* = 8)**	**Day 90** **(*n* = 3)**
**Adenocarcinoma** **(*n* = 3)**	21.6 (14.0-57.3) (*n* = 3)	36.8 (12.3-61.4) (*n* = 2)	11.6 (6.9-16.3) (*n* = 2)	5.2 (*n* = 1)
**Squamous cell carcinoma (*n* = 6)**	14.8 (7.6-65.5) (*n* = 6)	8.7 (5.6-12.6) (*n* = 3)	12.6 (5.1-26.7) (*n* = 4)	9.0 (6.6-11.4) (*n* = 2)
**Melanoma** **(*n* = 3)**	7.5 (0.6-17.6) (*n* = 3)	8.3 (2.9-13.7) (*n* = 2)	6.8 (2.8-10.7) (*n* = 2)	

Data are presented as median values (ranges); units are in cm^3^.

### CT perfusion imaging

A total of 30 CT perfusion examinations from 12 client-owned animals (11 dogs and 1 cat) were included in this study. All animals underwent CT perfusion imaging before receiving RT. Perfusion imaging was performed for 11 cases after RT, resulting in 18 follow-up examinations: 7 scans on day 0, 8 on day 30, and 3 on day 90 after RT completion. Two cases died before RT and day 0 after RT, respectively. Therefore, CT perfusion imaging was only available before RT for these cases.

During the CT perfusion procedure, the median heart rate was 140 bpm (range, 120-156 bpm) and the median systolic blood pressure was 145 mmHg (range, 100-180 mmHg). No adverse effects related to the contrast agent or anesthesia were observed in CT perfusion studies.

In color maps, the perfusion variables were successfully assessed. In 2 dogs, tumor mineralization caused localized voids in the FE map, but these did not compromise the evaluation of the lesion; therefore, all examinations were included in the analysis. In 4 scans from 2 dogs, contralateral parenchyma examination was not performed either due to the absence of contralateral parenchyma or inability to reliably identify normal tissue.

### CT perfusion variables before RT

Absolute perfusion variables before RT (*n* = 12) were as follows: BV 29.7 mL/100 mL (range, 5.7-156.4 mL/100 mL), BF 229.9 mL/min/100 mL (range, 19.6-817.2 mL/min/100 mL), and FE 39.5 mL/min/100 mL (range, 9.4-104.5 mL/min/100 mL). Relative ratios to contralateral parenchyma were 4.2 (range, 1.6-10.7) for rcBV, 2.5 (range, 0.5-8.6) for rcBF, and 1.5 (range, <0.1-3.1) for rcFE. Relative ratios to muscle were 4.2 (range, 1.5-14.8) for rmBV, 2.7 (range, 0.3-9.5) for rmBF, and 2.2 (range, 0.1-6.5) for rmFE.

Computed tomography perfusion variables were different across tumor types (Table 3). Melanoma generally showed higher BV, whereas adenocarcinoma had higher BF and all relative perfusion ratios including both rc and rm variables. In contrast, SCC showed lower rcBF, and rcBV and melanoma presented with lower rcFE. Relative to muscle, adenocarcinoma showed consistently higher perfusion ratios across all values, whereas SCC tend to have lower values.

**Table 3:** CT perfusion variables before radiation therapy according to head and neck tumor types.

**Tumor types**	**Absolute values**			**Relative to contralateral parenchyma (rc)**			**Relative to contralateral muscle (rm)**		
	**BV** **(mL/100 mL)**	**BF** **(mL/min/100 mL)**	**FE** **(mL/min/100 mL)**	**rcBV**	**rcBF**	**rcFE**	**rmBV**	**rmBF**	**rmFE**
**Adenocarcinoma (*n* = 3)**	27.3 (20.1-34)	293.1 (175.8-300.5)	47.1 (20.5-65.2)	4.5 (3.7-5.4)	4.2 (3.9-4.6)	1.7 (1.3-2.1)	5.7 (2.9-14.8)	5.7 (3.7-6.7)	2.5 (2.3-6.5)
**Squamous cell carcinoma (*n* = 6)**	27.0 (5.7-156.4)	215.9 (19.6-817.2)	49.7 (4.9-104.5)	1.9 (0.3-10.7)	1.5 (0.5-8.6)	1.6 (0.0-2.8)	1.6 (0.4-13.8)	1.3 (0.6-9.5)	2.0 (.1-4.0)
**Melanoma (*n* = 3)**	35.5 (33.2-53.0)	179.7 (166.9-471.0)	22.6 (9.4-70.5)	4.3 (1.6-5.5)	1.8 (1.7-2.1)	1.1 (1.0-1.8)	4.3 (1.5-7.2)	2.3 (2.2-6.5)	2.0 (1.9-4.3)

Data are presented as median values (ranges).

Abbreviations: BF = blood flow; BV = blood volume; FE = flow extraction.

### Association with CT perfusion variables before RT and treatment response

A total of 11 animals underwent RT with varying fractionation protocols, determined by tumor characteristics, clinician discretion, clinical condition, and client preference. Total prescribed radiation doses ranged from 24 to 42 Gy, with fractionation schedules varying from hypofractionated weekly regimens to conventional daily treatments. The most commonly used protocol was 8 Gy per fraction administered weekly for a total of 4 fractions (*n* = 4). Three dogs received 4.2 Gy per fraction for 10 daily fractions, and 1 dog was treated 3.2 Gy per fraction for 10 daily fractions. One dog was treated with 8 Gy per fraction for 3 fractions delivered on days 0, 7, and 21. Two dogs received a total dose of 36 Gy in 6 fractions of 6 Gy each, with one treated weekly and the other twice weekly.

**Figure 2:**
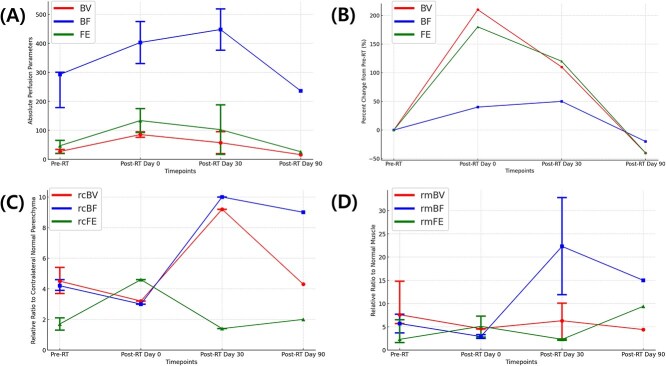
Changes in perfusion variables of adenocarcinoma over time. (A) Absolute values of perfusion variables including blood volume (BV), blood flow (BF), and flow extraction (FE). (B) Percent changes in absolute perfusion variables—BV, BF, and FE values—from pre-treatment values (pre-RT) values at each time point. (C) Relative ratio of lesion to contralateral normal parenchyma for BV (rcBV), BF (rcBF), and FE (rcFE). (D) Relative ratio of lesion to normal muscle for BV (rmBV), BF (rmBF), and FE (rmFE). Each graph displays three perfusion variables (BV, BF, and FE), represented in different colors. The x-axis represents four timepoints: pre-RT, post-RT day 0, post-RT day 30, and post-RT day 90. Dots and whiskers represent median values and range for each timepoint.

**Figure 3:**
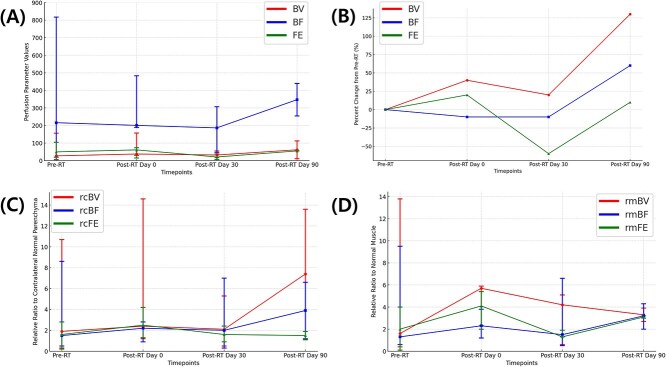
Changes in perfusion variables of squamous cell carcinoma over time. (A) Absolute values of perfusion variables including blood volume (BV), blood flow (BF), and flow extraction (FE). (B) Percent changes in absolute perfusion variables—BV, BF, and FE values—from pre-treatment values (pre-RT) values at each time point. (C) Relative ratio of lesion to contralateral normal parenchyma for BV (rcBV), BF (rcBF), and FE (rcFE). (D) Relative ratio of lesion to normal muscle for BV (rmBV), BF (rmBF), and FE (rmFE). Each graph displays three perfusion variables (BV, BF, and FE), represented in different colors. The x-axis represents four timepoints: pre-RT, post-RT day 0, post-RT day 30, and post-RT day 90. Dots and whiskers represent median values and range for each timepoint.

**Figure 4:**
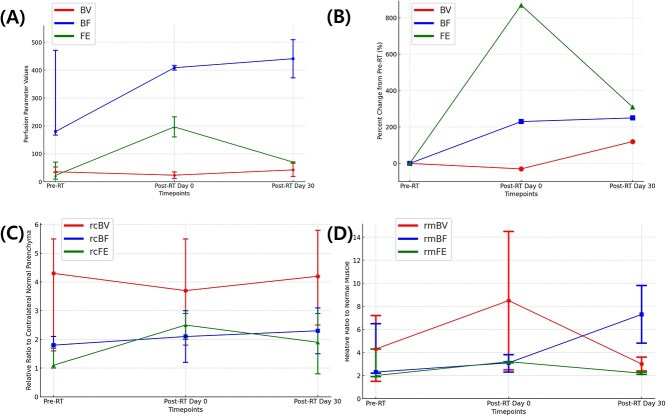
Changes in perfusion variables of melanoma over time. (A) Absolute values of perfusion variables including blood volume (BV), blood flow (BF), and flow extraction (FE). (B) Percent changes in absolute perfusion variables—BV, BF, and FE values—from pre-treatment values (pre-RT) values at each time point. (C) Relative ratio of lesion to contralateral normal parenchyma for BV (rcBV), BF (rcBF), and FE (rcFE). (D) Relative ratio of lesion to normal muscle for BV (rmBV), BF (rmBF), and FE (rmFE). Each graph displays three perfusion variables (BV, BF, and FE), represented in different colors. The x-axis represents four timepoints: pre-RT, post-RT day 0, and post-RT day 30. Dots and whiskers represent median values and range for each timepoint.

After treatment, cases with high perfusion variables before RT often showed greater tumor size reduction or sustained tumor regression over time, although statistical analysis was not conducted. These changes were represented in Figures 2-4, which showed the temporal changes in perfusion variables. In contrast, cases with low perfusion values before RT showed limited tumor size reduction or clinical deterioration. However, exceptions were observed, as some cases with high perfusion before RT demonstrated rapid tumor progression or metastases.

### Tumor size and CT perfusion variables after RT

Tumor volume generally decreased across all tumor types after RT, although the degree of response varied. On images after RT, the median tumor volume decreased to 12.3 cm^3^ (range, 2.9-61.4 cm^3^) on day 0 and continued to decline to 6.9 cm^3^ (range, 0.4-26.7 cm^3^) on day 30 and to 6.8 cm^3^ (range, 0.6-25.2 cm^3^) on day 90. Tumor volume changes of different tumor types at different time points are summarized in Table 2.

For adenocarcinoma, the tumor volume initially increased at day 0 after RT but showed a marked reduction at day 30 after RT and further decreased by day 90 after RT. Among the 3 adenocarcinoma dogs, 2 underwent imaging on day 0 and day 30 after RT, while only 1 was available for imaging on day 90 after RT. On imaging after RT, most perfusion variables including absolute values of BV, BF, and FE and rc variables (rcBV, rcBF, and rcFE) in adenocarcinomas showed initial increases after RT, reaching peak values at either day 0 or day 30 after RT, and subsequently declining by day 90 (Figure 2). The rmBV, rmBF also reflected this trend, peaking early after RT and decreasing at day 90. However, rmFE exhibited a distinct pattern, showing a continuous increase throughout the period after RT without a subsequent decline.

Among 6 SCC cases, 1 showed disease progression and was excluded from imaging after RT. Of the remaining 5, 2 were not imaged on Day 0 due to a short RT protocol, while another died before Day 30 and one more before Day 90 after RT. Additionally, 2 cases were not imaged on Day 90 after RT per owner request. Accordingly, perfusion imaging was performed in 3 scans on day 0, 4 scans on day 30, and 2 scans on day 90 after RT.

Tumor volume initially decreased on Day 0 after RT, showed a temporary increase by Day 30, and then declined again by Day 90. Variables in SCC generally increased on Day 0 after RT, decreased on Day 30, and then increased again on Day 90 (Figure 3). However, rcFE exhibited a different trend, rising on Day 0 after RT but declining continuously until Day 90. In the 2 cases that underwent imaging on Day 90 after RT, 1 exhibited tumor progression with rising all perfusion variables except for rmBV, while the other case, showing continuous tumor regression until Day 90, displayed decreased BV but increased other perfusion variables.

Of the 3 melanoma cases, 1 was lost to follow-up due to metastasis after RT and 2 cases was available for imaging through Day 30 after RT. Tumor volume increased initially on Day 0 after RT but subsequently decreased by Day 30. On imaging after RT, melanoma exhibited initial increase in most perfusion variables on day 0, except for BV and rcBV. By day 30 after RT, absolute perfusion variables, particularly BF, increased. While relative variables showed mixed trends, rcBF and rmBF continued to rise, whereas other relative variables fluctuated (Figure 4). For example, rcBV decreased initially at day 0 after RT, then increased by day 30, while rmBV, rcFE, and rmFE showed early increase on day 0 after RT but followed by declines at day 30.

### Association between CT perfusion and tumor size

Several perfusion variables, including p**Δ**BF, p**Δ**rcBF, rcBV, p**Δ**rcBV, and p**Δ**rmBV, appeared to correspond with changes in tumor volume (f**Δ*V***), length (f**Δ*l***), and height (p**Δ*h***). Among these, p**Δ**rmBV showed a consistent association with f**Δ*l***. Description of these changes are provided as supplementary data (Figure S1). These findings suggest that increases in rcBV or pΔBF is likely associated with tumor growth, while decrease in these variables might reflect tumor reduction. Additionally, an increase in pΔrmBV could predict a subsequent increase in tumor size on follow-up imaging. In contrast, no consistent pattern was observed between tumor size-related variables and either the absolute or normalized FE or its change ratios (pΔFE, fΔFE), suggesting that FE did not have a direct correlation with tumor size dynamics in this study.

Unlike height, volume, and length, tumor width did not demonstrate a consistent relationship with perfusion changes, suggesting that perfusion alterations are more reflective of height and length expansion rather than changes in tumor width.

## Discussion

This study evaluated the clinical role of CT perfusion imaging in assessing tissue perfusion across various tumor types and response to RT in dogs and cats with head and neck tumors. Tumor with higher BF and BV before RT generally showed greater reductions in tumor size after RT, suggesting that greater perfusion could be more radiosensitive. Additionally, relative perfusion values, such as rcBV and pΔBF, demonstrated closer correlation with tumor size changes than absolute values. These findings indicate their potential as functional imaging biomarkers for treatment response assessment. After RT, an early increase in perfusion variables was observed within the first 30 days, followed by a decline by day 90 in most tumors, consistent with previous findings in human studies.^22^^,^^24^ These results suggest that early perfusion changes might serve as potential indicators of treatment response.

Melanomas exhibited higher relative BV and BF values than SCC. This result is in contrast to a previous CT perfusion study in dogs with oral tumors, where SCC had higher perfusion than melanomas. These discrepancies could be attributed to tumor location, vascular remodeling, or differences in hypoxic adaptation. The higher perfusion observed in melanoma in our study might reflect the inherent aggressive nature of the tumors, potentially contributing to their increased metastatic potential.

Similarly, SCC in our study showed high perfusion variables than previously reported in dogs with SCC cases, where the median BV and BF before RT were of 10 mL/100 g and 107.6 mL/100 g/min, respectively.^3^ These differences might be related to tumor staging, microenvironmental variations, including tumor size, necrosis, and inflammatory response. Higher malignancy in SCC has been associated to increased microvascular density,^30^ which correlates with increased BF and BV.^18^^,^^31^

While most studies have reported that a higher BF and BV at the pre-treatment phase are associated with a favorable treatment response, some controversies remain.^9^^,^^21^^,^^22^^,^^25–27^^,^^32^ Our findings are somewhat consistent with the trend observed in previous study, where cases with relatively higher perfusion values tended to show better treatment responses, with tumor size reduction at the final follow-up. However, given the limited sample size and the presence of cases in which tumors progressed despite high perfusion values, drawing a definitive conclusion remains challenging. Additionally, differences in perfusion imaging protocols and methods between studies further limit direct comparisons. Therefore, while our results suggest a potential association between higher perfusion values and improved treatment response, further large-scale studies with standardized imaging protocols are required to validate these findings.

Regarding after RT, the dynamic changes in perfusion variables varied across tumor types, reflecting differences in vascular remodeling and radiation-induced tissue changes. In general, most tumor types exhibited an early increase in perfusion variables within the first 30 days after RT, consistent with studies suggesting transient radiation-induced hyperemia and inflammatoryresponses.^2^^,^^5^^,^^33^ The correlation between BF and treatment response after RT are reported in human and veterinary medicine, with early-phase perfusion changes being better predictable of treatment outcomes than late-phase changes. In dogs with nasal epithelial tumors, perfusion variables increased after 12 Gy irradiation as a part of 48 Gy RT protocol, even when tumor size was decreased in most cases.^2^

Adenocarcinoma cases in this study initially showed an increase in perfusion variables followed by a decline by day 90 and these cases demonstrated actual locoregional control, as evidenced by tumor size reduction at the final follow-up. Meanwhile, SCC showed a continuous increase in perfusion values at day 90 after RT compared to values before RT, including cases with clinical deterioration at follow-up. Therefore, based on these findings, our study results appear to align with patterns reported in human studies. Similarly, in a study including 15 human patients with head and neck SCC, the locoregional control group exhibited increased BF early in RT initiation, while the treatment failure group showed a decrease.^21^ Then, at late-phase, six weeks afterRT of median 70 Gy, both groups demonstrated a reduction in BV and BF. These findings suggest that an initial perfusion increase, followed by a decline, might indicate a favorable treatment response and locoregional control.^3^ In contrast, sustained rise in perfusion might indicate tumor progression rather than successful locoregional control.

Among the perfusion variables measured in this study, BF and BV more accurately reflected treatment response than FE, as they showed a stronger association with tumor size, whereas FE exhibited fluctuating or steady patterns. This finding aligns with human medicine, where BF and BV are recognized as direct indicators of tumor vascular characteristics, angiogenesis, and metabolic demands, influencing tumor growth rates through oxygen and nutrient delivery.^14–17^^,^^20^

Additionally, relative perfusion variables better reflected clinical status than absolute variables, with relative or normalized values (rcBV and pΔBF) showing associations with tumor size and its change ratio. Consequently, relative variables (rc or rm) were considered more closely linked to tumor size changes than absolute variables, supporting previous studies that emphasize the clinical significance of normalized perfusion values.^34–36^ Notably, pΔrmBV was identified as a key variable for predicting future tumor size changes, suggesting that these variables could aid in treatment decision-making and response prediction.

Given the heterogeneous perfusion responses across tumor types, tumor-specific perfusion profiling could aid in RT planning and patient stratification. For example, highly perfused tumors such as adenocarcinoma and SCC might benefit from conventional RT, while hypoperfused tumors might require alternative therapeutic strategies, such as hypoxia-modifying agents or dose escalation protocols.

Unlike tumor height, length, and volume, the tumor width did not show a correlation with perfusion changes. This finding might be partly attributed to the anatomical constraints, as most tumors in this study were located in confined spaces, where lateral expansion is limited by rigid structures like bone. These spatial limitations might have restricted transverse tumor growth, resulting in less pronounced changes in width. This finding presented the importance of incorporating tumor location and anatomical context when evaluating perfusion-based changes in tumor dimensions. These findings should be interpreted with consideration of several limitations. First, this study included only a small sample size. Second, variations in CT perfusion methodology might have contributed to discrepancies. A previous study that analyzed perfusion variables in dogs with nasal tumors—7 adenocarcinomas and 4 sarcomas—using 3 different perfusion software programs reported BF values ranging from 51 to 72 mL/min/100 g and BV values between 6.2 and 7.7 mL/100 g, demonstrating considerable variability even within the same case. Standardized imaging protocols are necessary to improve reproducibility and reliability across studies. Third, inter-species differences between dogs and cats were not accounted for in this study. Although the single SCC case in a cat showed perfusion variables that were comparable to those observed in both previous dog studies and the current dog cohort, physiological and hemodynamic difference might still exist. Moreover, when measuring relative perfusion variables, the comparison was made not only to the tumor hemodynamics but also to those of normal tissues and muscle, which likely caused an additional limitation. As such, the inclusion of the cat in this study should be interpreted cautiously. Fourth, differences in the clinical status, as well as the influence of reperfusion or pseudoprogression were not fully evaluated. While tumor perfusion variable changes were measured over time, it was challenging to accurately distinguish between perfusion changes related to tumor remission and progression within the same tumor group. Additionally, the animals received different prescription dose and fractionation schedules, which might have influenced perfusion responses. Finally, the effects of radiosensitizers or other medications were not analyzed, which could have influenced the perfusion dynamics observed in this study.

This study demonstrated the potential utility of CT perfusion imaging in evaluating tumor perfusion and predicting therapeutic response in veterinary oncology. Normalized blood volume before RT (rcBV) and the ratio of BF before to after RT showed the association with tumor size reduction. Additionally, changes in muscle-normalized BV provide useful information for guiding treatment decisions. These findings suggest their value as functional imaging biomarkers.

## Supplementary Material

aalaf025_Supplementary_Figure_1

## Off-label antimicrobial declaration

The authors declare no off-label use of antimicrobials.

## Institutional animal care and use committee or other approval declaration

Approved by Seoul National University Institutional Animal Care and Use Committee (IACUC), approval number IACUC-SNU-230501-1-1.

## Abbreviations

BF: blood flow
BV: blood volume
CT: computed tomography
CTV: clinical target volume
FE: flow extraction
FLAIR: fluid-attenuated inversion recovery
FOV: field of view
fΔ: post-RT change ratio of following examination
GTV: gross tumor volume
OAR: organs at risk
pΔ: post-RT change ratio compared to the previous examination
post-RT: after the completion of radiotherapy
pre-RT: before radiotherapy
PTV: planning target volume
rc: relative to contralateral normal tissue
RECIST: Response Evaluation Criteria in Solid Tumors
rm: relative to normal muscle
RT: radiotherapy
ROI: region of interest
SCC: squamous cell carcinoma
T1W: T1-weighted
T2W: T2-weighted
