# Supplementary material for: Computed tomography perfusion in predicting radiation therapy response in dogs and a cat with head and neck tumors
Source: J Vet Intern Med. 2026 Jan 21;40(1):aalaf025. doi: 10.1093/jvimsj/aalaf025 (PMC12881964; doi:10.1093/jvimsj/aalaf025)
Supplement: aalaf025_Supplementary_Figure_1 [file aalaf025_supplementary_figure_1.docx]

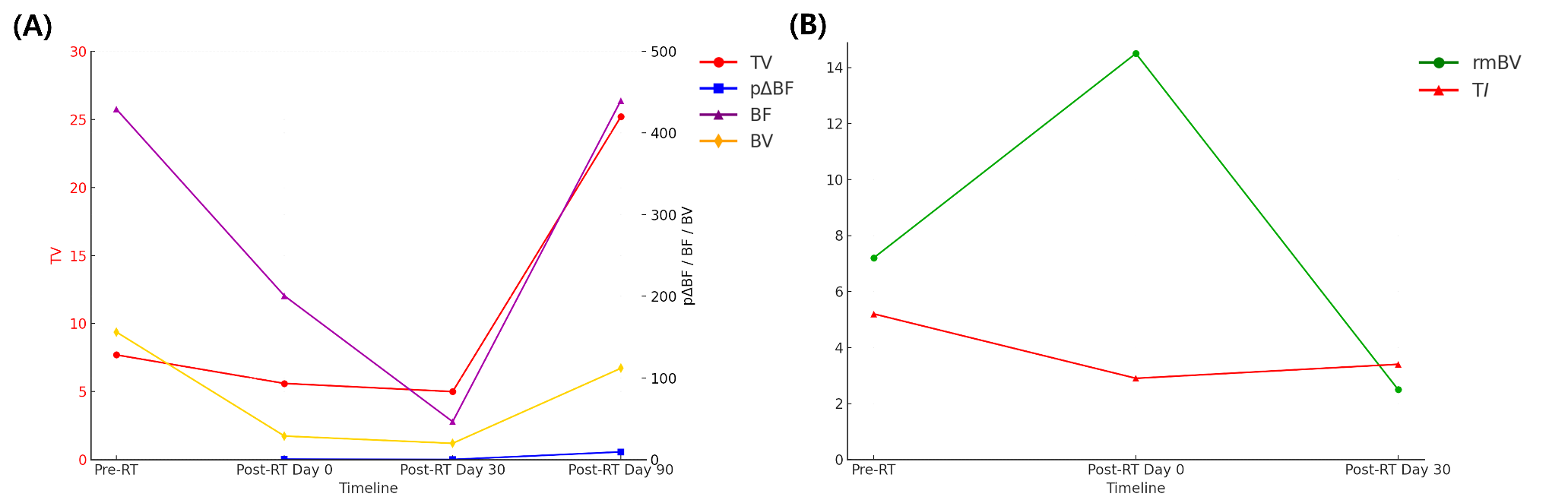


**Supplementary Figure S1.** Association between perfusion parameters and tumor size parameters. (A) Perfusion parameters, including blood volume (BV; yellow), blood flow (BF; purple), and the post-RT change ratio of BF compared with the previous examination (pΔBF; blue), exhibited temporal patterns similar to changes in tumor volume (TV; red).(B) The relative ratio of lesion to normal muscle for BV (rmBV; green), demonstrated an increase that preceded the change in tumor length (Tl; red), suggesting its potential role as a predictive marker.
